# Surface Electromyography and Electroencephalogram-Based Gait Phase Recognition and Correlations Between Cortical and Locomotor Muscle in the Seven Gait Phases

**DOI:** 10.3389/fnins.2021.607905

**Published:** 2021-05-21

**Authors:** Pengna Wei, Jinhua Zhang, Baozeng Wang, Jun Hong

**Affiliations:** The Key Laboratory of Education Ministry for Modern Design and Rotor-Bearing System, School of Mechanical Engineering, Xi’an Jiaotong University, Xi’an, China

**Keywords:** electroencephalogram, surface electromyography, gait phases, pattern recognition, time–frequency cross mutual information

## Abstract

The classification of gait phases based on surface electromyography (sEMG) and electroencephalogram (EEG) can be used to the control systems of lower limb exoskeletons for the rehabilitation of patients with lower limb disorders. In this study, the slope sign change (SSC) and mean power frequency (MPF) features of EEG and sEMG were used to recognize the seven gait phases [loading response (LR), mid-stance (MST), terminal stance (TST), pre-swing (PSW), initial swing (ISW), mid-swing (MSW), and terminal swing (TSW)]. Previous researchers have found that the cortex is involved in the regulation of treadmill walking. However, corticomuscular interaction analysis in a high level of gait phase granularity remains lacking in the time–frequency domain, and the feasibility of gait phase recognition based on EEG combined with sEMG is unknown. Therefore, the time–frequency cross mutual information (TFCMI) method was applied to research the theoretical basis of gait control in seven gait phases using beta-band EEG and sEMG data. We firstly found that the feature set comprising SSC of EEG as well as SSC and MPF of sEMG was robust for the recognition of seven gait phases under three different walking speeds. Secondly, the distribution of TFCMI values in eight topographies (eight muscles) was different at PSW and TSW phases. Thirdly, the differences of corticomuscular interaction between LR and MST and between TST and PSW of eight muscles were not significant. These insights enrich previous findings of the authors who have carried out gait phase recognition and provide a theoretical basis for gait recognition based on EEG and sEMG.

## Introduction

Human locomotor disorder seriously affects the quality of life. Nontraumatic gait disorder is caused by brain damage and is a feature of many neurological disorders such as stroke, cerebral palsy, and Parkinson’s disease (Louie and Eng, 2016; Ziegler et al., 2018). Surface electromyography (sEMG)-based rehabilitative devices or robots have been developed for neurological injury rehabilitation of lower limb functions (Veneman et al., 2007; Banala et al., 2008). The classification results of gait phases from sEMG can be used to control the gait of lower limb exoskeletons for the rehabilitation of patients with lower limb disorders (Joshi et al., 2013). The human gait cycle is divided into stance and swing phases (Taborri et al., 2016). In this study, the stance phase is then subdivided into loading response (LR), mid-stance (MST), terminal stance (TST), and pre-swing (PSW). Similarly, the swing phase is divided into the initial swing (ISW), mid-swing (MSW), and terminal swing (TSW). More information about gait partitioning methods is available elsewhere in the literature (Taborri et al., 2016).

However, sEMG signals change due to muscle fatigue and sweat. Patients will suffer from muscle fatigue after training. Therefore, gait phase recognition methods only using sEMG signals are limited, meaning they cannot identify all seven gait phases. For instance, Li et al. (2016) utilized sEMG signals to recognize five gait phases, while Wei et al. applied sEMG and kinematic data from both legs to recognize five gait phases (Wei et al., 2018). However, electroencephalogram (EEG) signals will counteract these shortcomings (susceptible to fatigue and sweating) of sEMG. The cortex is activated during walking and EEG signals will enrich the gait information for gait prediction. EEG and sEMG signals, generated before movement, can be used to predict gait (Gao et al., 2018; Ziegler et al., 2018). Nevertheless, the theoretical basis of gait phase recognition based on EEG combined with sEMG has not been researched.

Human bipedal walking is an automatic activity. It includes top-down pathways (from the brain toward the spinal cord and periphery) to generate a motor action, while it also includes feedback from the periphery to the brain to correct the motion. Several research groups have observed significant cortical activation (for example, in the premotor, supplementary motor, and primary sensorimotor regions) during walking (la Fougère et al., 2010; Bradford et al., 2016; Artoni et al., 2017). In 2019, Jensen and colleagues found that the motor cortex contributes to both ankle plantar flexor muscle activity and forward propulsion during gait (Jensen et al., 2019). The authors of recent studies have found that the gait phase is associated with cortical activity modulations (Wagner et al., 2012, 2014; Bradford et al., 2016). However, corticomuscular connectivity remains unclear during walking.

Various researchers have used either coherence or correlation analysis methods to measure corticomuscular coherence (Artoni et al., 2017; Jensen et al., 2019), such as Jensen et al. who performed frequency domain analysis of the correlation between EEG–EMG and EMG–EMG. However, coherence and correlation analysis are suitable for linear signals, while both EEG and sEMG signals are nonlinear (Popivanov and Dushanova, 1999). The nonlinear characteristics include point-wise correlation dimension, Kolmogorov entropy, and largest Lyapunov exponents as functions of time. The time–frequency cross mutual information (TFCMI) method can be utilized to calculate mutual information between two time–frequency domain signals (Lu et al., 2011; Anmin Gong et al., 2018). The TFCMI method integrates the time and frequency components of the signal, then the nonlinear correlation calculation method is used to estimate the similarity of multichannel physiological signals. We used TFCMI to estimate the interaction between EEG and sEMG channels during walking.

In this study, we firstly used sEMG, EEG signals, and 3D motion trajectory data (Taborri et al., 2016) for lower limbs to predict all seven gait phases. The robustness of sEMG and EEG-based neural interface was analyzed because people cannot always walk at a fixed speed (Marchal-Crespo and Reinkensmeyer, 2009; Tefertiller et al., 2011).

Therefore, we predict the gait phases at three speeds. Secondly, we calculated TFCMI values between beta-band EEG and sEMG channels in the seven gait phases to research the theoretical basis of gait phase recognition based on EEG and sEMG (Oliveira et al., 2018; Li et al., 2019).

## Materials and Methods

### Participants and Ethical Approval

Nine healthy participants (seven males and two females, aged 23–26; body weight: 62 ± 10 kg; height: 171 ± 6 cm) were recruited from Xi’an Jiaotong University. All study procedures performed were approved by the Institutional Review Board of Xi’an Jiaotong University and carried out according to the Helsinki Declaration of 1975.

### Experimental Device

**FIGURE 1:**
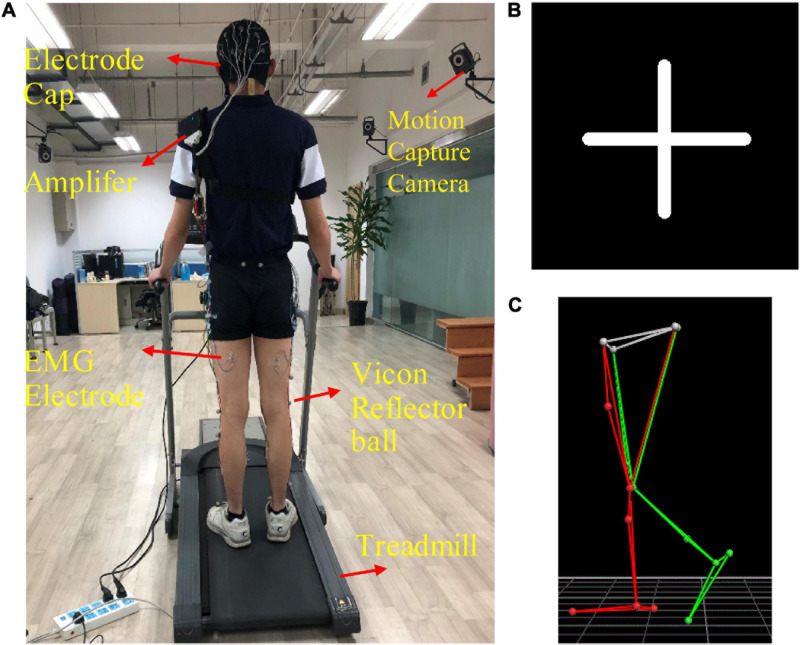
**(A)** The experimental device, **(B)** the cross symbol, and **(C)** the lower limb model in the motion capture system. The 16 positions of the Vicon reflector balls are the anterior superior iliac, posterior superior iliac, thigh, knee, tibia, ankle, heel, and toe, bilaterally.

The custom-designed equipment used in this experiment included a treadmill, an electrode cap, an amplifier with a built-in three-axis acceleration sensor, eight sEMG electrodes, 10 motion capture cameras, and 16 Vicon reflector balls, as shown in Figure 1A. We utilized a 32-channel LiveAmp Cap with multitrodes to record EEG and sEMG signals during treadmill walking. This LiveAmp Cap was a customized, wireless, lightweight, wearable device, meaning it caused minimum disturbance to the participants’ movement while EEG and sEMG were recorded. A gaze screen with a cross symbol was used to focus the participants, as shown in Figures 1B,C shows the lower limb model in a motion capture system. All participants walked on the treadmill using their typical walking style. Participants choose a comfortable treadmill speed of 2.0 km/h during the experiment. The speeds of 1.4 and 2.6 km/h were also selected to simulate slow and fast walking speeds in everyday life.

### Data Collection

All participants walked on a treadmill at three speeds (1.4, 2.0, and 2.6 km/h), respectively, in fifteen 30-s time blocks. There was a break between any two trials. Participants were asked to walk as usual, as well as to relax and minimize eye blinks, head rotation, and swallowing during the study. Participants were also asked to fix their gaze to the cross symbol, as shown in Figure 1B. The experiments were conducted in a quiet room.

EEG and sEMG signals and lower limb trajectory data were simultaneously recorded. Twenty-four-channel EEG signals (Fp1, Fp2, F3, F4, C3, C4, P3, P4, O1, O2, F7, F8, T7, T8, P7, P8, Fz, Cz, Pz, Oz, M1, M2, FPz, and VEOG) and eight muscles [biceps femoris (BF), vastus medialis (VM), tibialis anterior (TA), and gastrocnemius medialis (GM), bilaterally] of the lower limb sEMG signals were collected by the 32Ch LiveAmp Cap at a sampling rate of 500 Hz. Twenty-four unipolar channels were collected in the cap, and electrodes for eight bipolar sEMG were recorded at the BF, VM, TA, and GM. These muscles were selected as they are related to the entire gait cycle (Perry and Davids, 1992; Petersen et al., 2012). Locations for sEMG electrodes were selected based on the SENIAM guidelines^1^ (Artoni et al., 2017), while EEG electrodes were placed following the international 10–20 electrode system. The impedance of EEG and sEMG electrodes was kept under 20 kΩ throughout the experiment (Gwin et al., 2011).

Signals were amplified using a wireless LiveAmp amplifier (Brain Products Inc., Gilching, Germany). The track of 16 positions of the lower limbs was acquired by 16 reflective markers, using a 10-camera motion capture system Nexus 2.6 (VICON T40S, United Kingdom) at a sampling rate of 100 Hz. Reflective markers’ positions are presented in Figure 1C. The motion capture system was synchronized with the wireless LiveAmp amplifier *via* the LiveAmp sensor and trigger extension. 3D marker data were resampled and aligned to EEG and sEMG data using a MATLAB (MathWorks Natick, MA, United States) script, based on the EEGLAB toolbox (Delorme and Makeig, 2004) before further preprocessing.

### Data Processing

#### EEG and sEMG Data Processing

Raw EEG data were preprocessed using Brain Vision Analyzer 2.1 (Brain Products Inc., Gilching, Germany) and a MATLAB (MathWorks, Natick, MA, United States) script based on the EEGLAB toolbox, thus minimizing motion and other artifacts (Kline et al., 2015). The raw signal processing flow can be seen in Supplementary Figure 1. The default reference position of the electrode cap is FCz. However, FCz was too close to other EEG electrodes. Therefore, raw data were re-referenced to the mastoid, and the mean of the left and the right mastoid signal was calculated as a new reference. The EEG, EOG, and three-axis acceleration channels were selected. EEG signals were then high-pass filtered using a zero-phase 0.5 Hz cutoff, second-order Butterworth filter, and low-pass filtered using a zero-phase 50 Hz.

The EEG and sEMG data were resampled to 1,000 Hz and independent component analysis (ICA) (Sun et al., 2005) was used to decompose EEG signals into many independent components (ICs). The ICs which most correlated to lateral and vertical eye movement were marked and removed. sEMG signals were passed through an elliptic bandpass filter of 30–450 Hz bandwidth, while an FIR least-square bandstop notch filter of 50 Hz was used to remove low and high frequencies and residual line noise from raw sEMG signals. Wavelet denoising technology was used to remove noise in sEMG signals. The basic functions of the wavelet we adopted were “wden” and “db4.” EEG and sEMG channels with obvious artifacts were removed following a visual inspection.

#### Gait Cycle Segmentation and Gait Phase Segmentation

**FIGURE 2:**
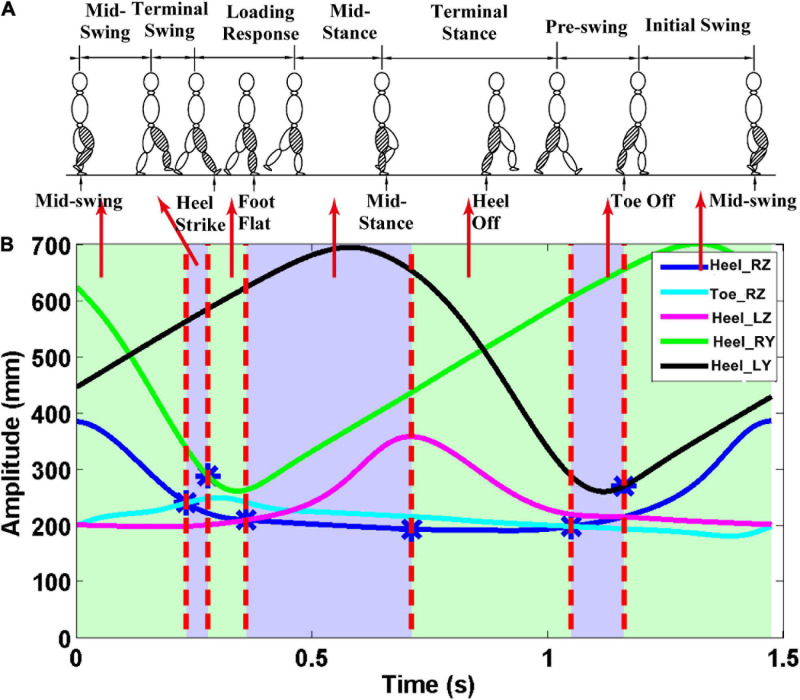
Gait phase division results. The *z*-direction corresponds to the vertical direction, and the *y*-direction is the anteroposterior. **(A)** Seven gait phases of the gait cycle; **(B)** trajectory data. The first vertical red line—the first intersection of Heel_RZ and Toe_RZ. The second vertical red line—the point with the largest difference between Heel_RY and Heel_LY. The third vertical red line—the first intersection of the Heel_RZ and Heel_LZ. The fourth vertical red line—the maximum point of the Heel_LZ. The fifth vertical red line—the second intersection of the Heel_RZ and the Toe_RZ. Finally, the sixth vertical red line—the point with the smallest difference between the Heel_RY and the Heel_LY.

3D marker data from the five positions were used to divide the gait cycle during treadmill walking. The *z*-direction corresponds to the vertical direction, the *y*-direction is the anteroposterior. The *z*-direction trajectory of the right heel with a more obvious periodicity of the gait cycle was applied to divide EEG and sEMG signals into single gait cycles (see Supplementary Figure 2). The seven gait phases are demarcated according to 3D marker data from the five positions (Perry and Davids, 1992; Ryu and Kim, 2014). The MSW is the period until the right foot is horizontal [vertical position of the right heel (Heel_RZ) equal to that of the right toe (Toe_RZ)] after ISW. TSW is the period until the distance between the left and right feet is the farthest at the first time[that is the first largest difference between the anteroposterior displacement of the right heel (Heel_RY) and that of the left heel (Heel_LY)] after the MSW. LR is the period until the right leg is being apart from land [vertical position of the right heel (Heel_RZ) equal to that of the left heel (Heel _LZ)] after TSW. MST is the period until the heel of the right leg is at the highest position after LR. TST is the period until the right foot is horizontal again after MST. PSW is the period until the distance between the left and right feet is the smallest after TST. ISW is the period until the end of the gait cycle. The gait phase segmentation result can be seen in Figure 2.

#### Feature Extraction

Feature extraction is a technique used to draw representation information from preprocessed input data. To analyze sEMG and EEG signals, feature extraction methods tend to include the time domain (TD), frequency domain (FD), and time–frequency (TFD) domain (Asghari Oskoei and Hu, 2007; Phinyomark et al., 2017; Martín-Clemente et al., 2018). In this study, slope sign change (SSC) and mean power frequency (MPF) were utilized to classify the seven gait phases. SSC is a time domain feature that also reflects signal frequency information. MPF is a mean frequency that is expressed as the sum of sEMG and EEG power frequency, divided by the total sum of the spectrum intensity. These extract features were combined and inputted to the library for support vector machine (LIBSVM) for classification. Three feature sets were combined. They were the SSC and MPF features of sEMG ([7 × 100, 8 × 2]), the SSC and MPF features of sEMG and the SSC feature of EEG ([7 × 100, (8 + 21) × 3]), the SSC and MPF features of sEMG, and the MPF feature of EEG ([7 × 100, (8 + 21) × 3]). In brackets are the feature set dimensions, 7 is the seven gait phases, 100 is the number of the feature values, 8 is the number of the sEMG channels, 21 is the number of the EEG channels, and 2 and 3 are the number of features (such as SSC of sEMG, SSC of EEG, etc.). Finally, two-thirds of the combined feature sets as training dataset were inputted to the support vector machine (SVM) to train the classifier. Then, one-thirds of the combined feature sets as testing dataset were inputted to the trained classifier to classify the seven gait phases. SVM parameters were optimized using the particle swarm optimization (PSO) method.

#### Time–Frequency Analysis Using TFCMI

TFCMI values were calculated to estimate the time–frequency correlation between EEG and sEMG channels. EEG and sEMG signals were normalized due to their large power difference before TFCMI was calculated. The calculation process can be seen below.

Morlet wavelet transformation, which contains both time and frequency domain information, was utilized to transform the EEG signal into the time–frequency domain (Vialatte et al., 2007). Time–frequency power maps of each channel for beta (16–25 Hz) data were created. Therefore, the two maps were 10 × 2,000 and 16 × 2,000, respectively. Ten and 16 represent the frequency from 16 to 25 Hz and 30 to 45 Hz, while 2,000 represents the sample points. The mean of beta-band powers was calculated, respectively, before two 1 × 2,000 power curves were created in each channel.

Cross mutual information (CMI) between any two channels was calculated using the mean power signals. CMI maps were created by computing the entropy and mutual information, which can be expressed as:

$$\text{H}\,\left({\text{F}}_{i}\right)=-\sum_{b=1}^{40}\text{p}\,\left({\text{F}}_{i,b}\right)\,\log_{2}\text{p}\,\left({\text{F}}_{i,b}\right)$$

$$\begin{array}{c} \text{TFMI}\,\left({\text{F}}_{i},{\text{F}}_{j}\right)=\text{H}\,\left({\text{F}}_{i}\right)+\text{H}\,\left({\text{F}}_{j}\right)-\text{H}\,\left({\text{F}}_{i},{\text{F}}_{j}\right)\\ =\sum_{b=1}^{40}\text{p}\,\left({\text{F}}_{i,b},{\text{F}}_{j,b}\right)\,\log_{2}\frac{p\,\left(F_{i,b},F_{j,b}\right)}{p\,\left(F_{i,b}\right)\,p\,\left(F_{j,b}\right)} \end{array}$$

where **H**(**F**_*i*_) denotes entropy, **F**_*i*_ is the mean power signals at the *i*th channel, **p**(**F**_*i*,*b*_) is the probability density function (PDF) of **F**_*i*_, **p**(**F**_*i*,*b*_,**F**_*j*,*b*_) is the joint probability density function (JPDF) of **F**_*i*_, while **F**_*j*_*b* = 1,2,⋯,40 is the bin number of the histogram used to construct the approximated PDF. Some 40 bins were selected based on both previous research and our data (Fraser and Swinney, 1986; Tamburri et al., 2002).

Finally, TFCMI values between any two channels were obtained to create a 31 × 31 (23 EEG and 8 sEMG channels) TFCMI map (Supplementary Figure 3A). Each entry of the 31× 31 matrices is the value of mutual information from TF power between a pair of channels. TFCMI values from the *i*th to the *j*th as well as the *j*th to the *i*th are the same. Therefore, the TFCMI map is symmetrical. Since TFCMI values were normalized, the diagonal TFCMI value (self-relevance) is equal to one. TFCMI values from the 13 EEG (lower limb movement-related electrodes: F3, F4, C3, C4, P3, P4, F7, F8, P7, P8, Fz, Cz, and Pz) and eight sEMG channels were extracted from the 31 × 31 matrices. We then calculated the mean of TFCMI values between 13 EEG and 8 sEMG channels in all trials, which were illustrated as a 13-channel topographic map. The map of mean coupling strength from the Oz channel to all EEG channels as well as the Fpz channel to all EEG channels can be seen in Supplementary Figure 3B.

## Results

### Gait Phase Recognition Results

It should be noted that the sEMG data of two participants are unavailable due to surface electrode malfunction. Therefore, this analysis is based on seven participants. Gait phase recognition was based on the SSC and MPF features of the sEMG and EEG signals. For easy understanding, we define the SSC and MPF features of sEMG as case 1, define the SSC and MPF features of sEMG and the SSC feature of EEG as case 2, and define the SSC and MPF features of sEMG and the MPF feature of EEG as case 3. Firstly, we compared gait phase recognition based on case 1 and case 2. Secondly, gait phase recognition using case 2 was compared with recognition using case 3.

#### Difference Between sEMG and EEG Signal Features

**FIGURE 3:**
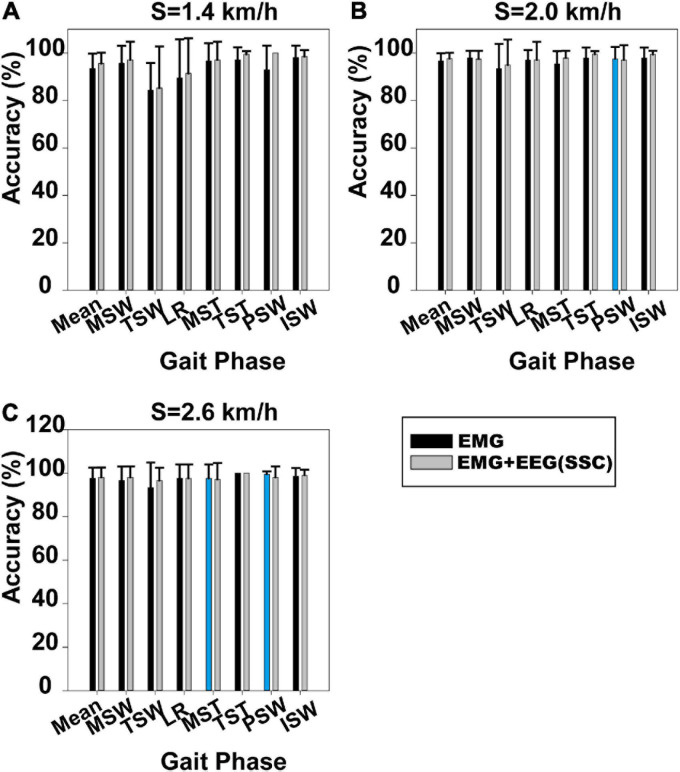
Gait phase recognition results based on case 1 and case 2. **(A)** Walking speed 1.4 km/h, **(B)** walking speed 2.0 km/h, and **(C)** walking speed 2.6 km/h. The blue column indicates that the results based on case 1 are better than those on case 2.

Gait phase identification based on case 1 and case 2 is shown in Figure 3. Gait phase identification in the seven gait phases and the three speeds (1.4, 2.0, and 2.6 km/h) can be seen in Figures 3A–C, respectively. Figure 3A demonstrates that the mean accuracy of recognition of the seven gait phases using case 1 and case 2 was 93.47 and 95.58%. Also, the standard deviation (SD) decreased from 0.062 to 0.045. The mean accuracy has increased when the SSC of EEG was applied. The same can be observed in Figures 3B,C. In Figure 3B, the mean accuracy of identifying the seven gait phases increased from 96.69 to 97.63%, while the SD decreased from 0.032 to 0.025. In Figure 3C, the mean accuracy of identifying the seven gait phases increased from 97.62 to 98.10%, while the SD decreased from 0.048 to 0.044.

The Mann–Whitney *U*-test was used to analyze whether the difference in gait phase recognition between case 1 and case 2 was significant. We found that the difference between case 1 and case 2 was significant at 1.4 km/h (*p* = 0.034), but not at 2.0 km/h (*p* = 0.244) and 2.6 km/h (*p* = 0.746). The results for each participant based on case 1 and case 2 can be seen in Supplementary Figures 4–6.

#### Difference Between the EEG Features of the SSC and MPF

**FIGURE 4:**
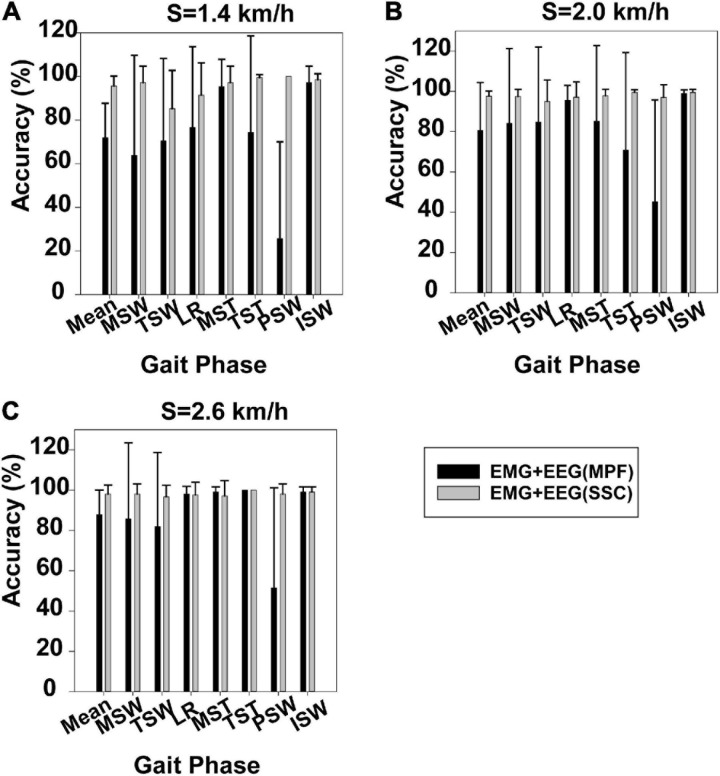
Gait phase recognition based on case 2 and gait phase recognition based on case 3. **(A)** Walking speed 1.4 km/h, **(B)** walking speed 2.0 km/h, and **(C)** walking speed 2.6 km/h.

Figure 4 displays gait phase recognition based on case 2 and gait phase recognition based on case 3. Gait phase recognition in the seven phases at three speeds (1.4, 2.0, and 2.6 km/h) can be seen in Figures 4A–C, respectively. These figures demonstrate that the mean accuracy of recognition of the seven gait phases decreased from 95.58 to 71.90%, 97.63 to 80.63%, and 98.10 to 87.89%, respectively, while the SD increased from 0.045 to 0.158, 0.025 to 0.23, and 0.044 to 0.121, respectively, when the SSC of EEG was replaced by the MPF of EEG. Some gait phase recognition results based on case 3 were below 60%, for example, PSW in Figures 4A–C. Using the Mann–Whitney *U*-test, we found that the gait phase recognition difference between the two sets was significant at 1.4 km/h (*p* = 0.003) but not significant at 2.0 km/h (*p* = 0.092) and 2.6 km/h (*p* = 0.123).

### Time–Frequency Analysis of EEG and sEMG Signals in Seven Gait Phases Using TFCMI

**FIGURE 5:**
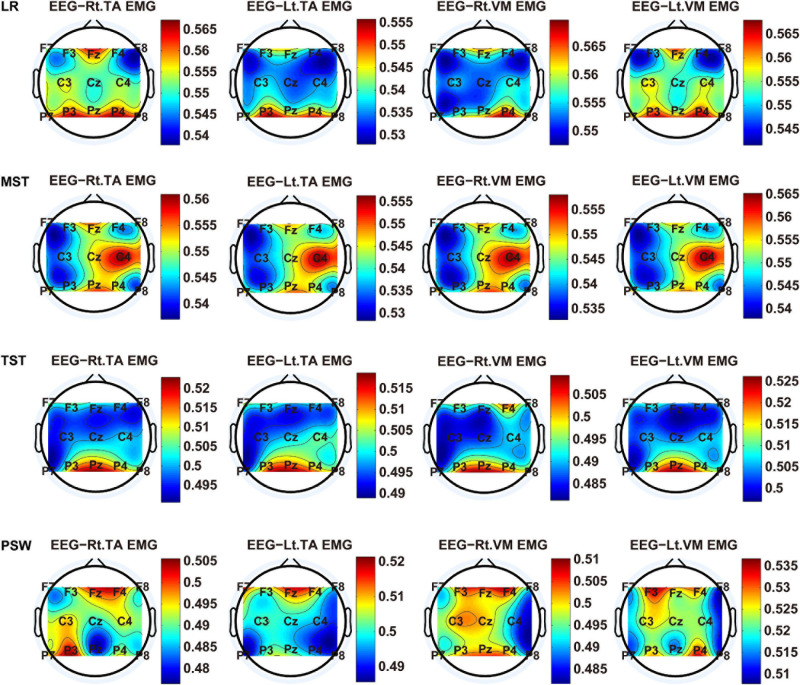
The TFCMI topography between EEG (beta band) and sEMG (TA and VM) of the stance phase (LR, MST, TST, and PSW).

**FIGURE 6:**
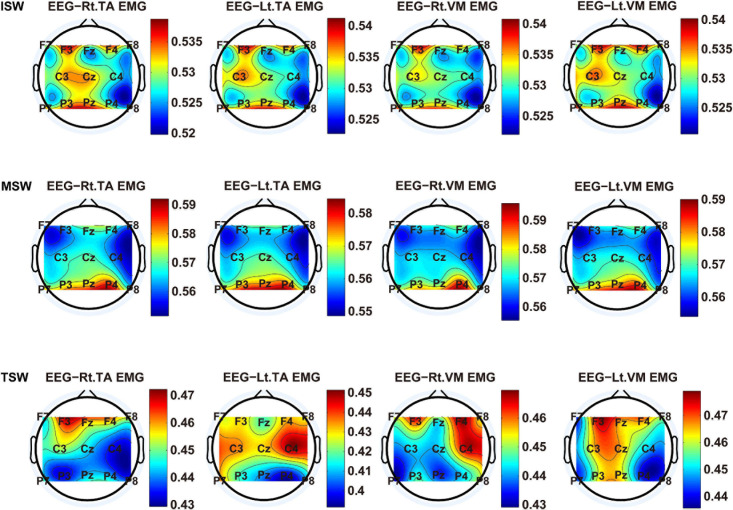
The TFCMI topography between EEG (beta band) and sEMG (TA and VM) of the swing phase (ISW, MSW, and TSW).

**FIGURE 7:**
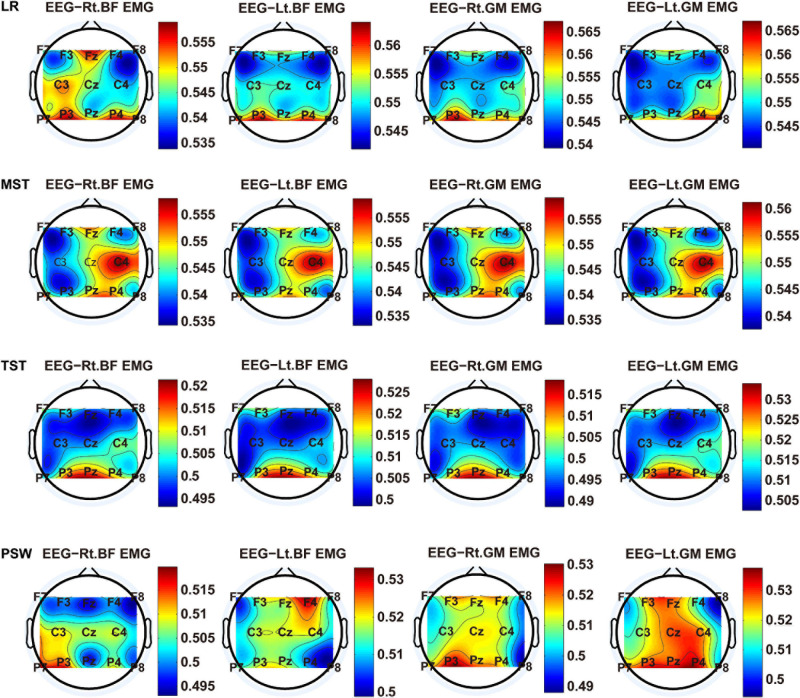
TFCMI topography between EEG (beta band) and sEMG (BF and GM) of the stance phase (LR, MST, TST, and PSW).

**FIGURE 8:**
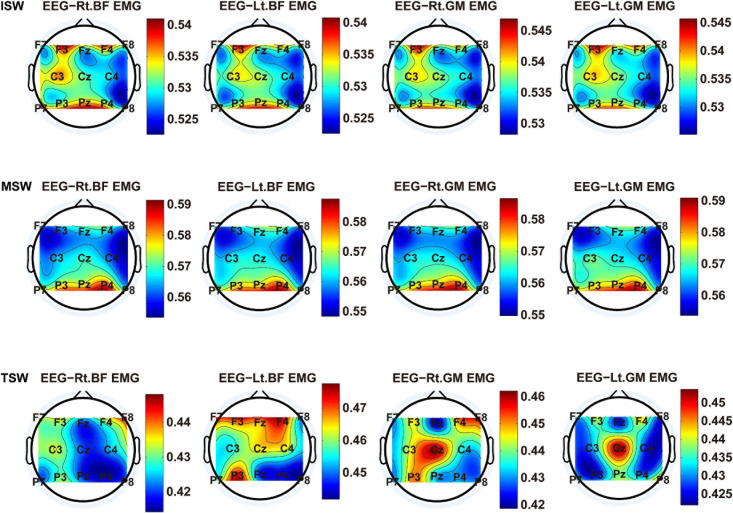
TFCMI topography between EEG (beta band) and sEMG (BF and GM) of the stance phase (ISW, MSW, and TSW).

We carried out a time–frequency analysis of the EEG and sEMG signals using TFCMI at a comfortable speed (2.0 km/h). TFCMI topography between EEG data (beta band) and sEMG can be seen in Figures 5–8. The results in Figures 5, 6 are based on the sEMG channels of the right (Rt.) and left (Lt.) TA as well as the right and left VM. Results in Figures 7, 8 are based on the sEMG channels of BF and GM of both legs. In Figures 5–8, we can observe that the distribution of TFCMI values of eight topographies was different in PSW (*p* < 0.05) and TSW (*p* < 0.05).

**FIGURE 9:**
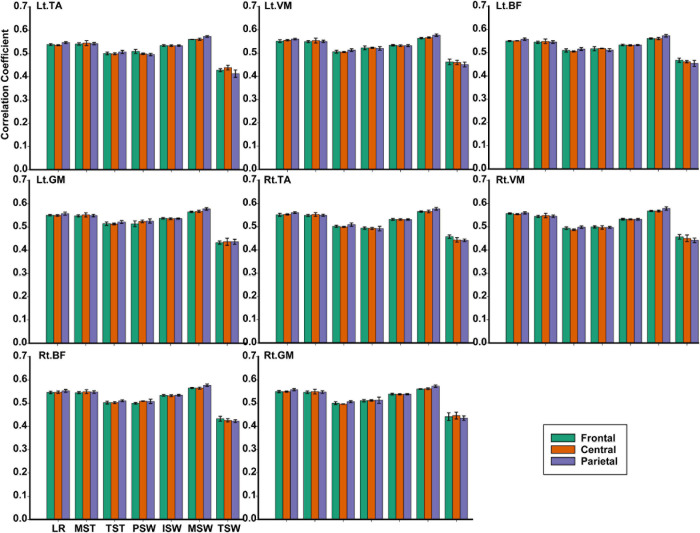
The TFCMI values of the frontal, central, and parietal lobes in seven gait phases for eight muscles.

TFCMI values of the frontal, central, and parietal lobes were calculated and can be seen in Figure 9. One-way ANOVA was utilized to explore whether the difference between TFCMI values for the seven gait phases of eight muscles was significant. The results showed that the difference between TFCMI values for the seven gait phases of each muscle was significant (*p* < 0.05). Multiple comparisons were used to explore which two phases of the eight muscles differed significantly. The gait phases with no significant difference can be seen in Table 1 due to the fact that there were too many phases with significant differences.

**TABLE 1:** Multiple comparison results of the seven gait phases for the beta band.

**Muscle**	**Gait phases**	***p*-value**
Lt. TA	LR and MST	0.688
	TST and PSW	0.802
Lt. VM	LR and MST	0.075
Lt. BF	TST and PSW	0.126
Lt. GM	LR and MST	0.214
	TST and PSW	0.172
Rt. VM	TST and PSW	0.135
Rt. BF	LR and MST	0.475
	TST and PSW	0.763
Rt. GM	LR and MST	0.095

## Discussion

Gait phase recognition based on case 2 was better than recognition based on case 1 and case 3, as can be seen in Figures 3, 4. The use of near-infrared spectroscopy (NIRS) (Miyai et al., 2001) and functional magnetic resonance imaging (fMRI) (Cunnington et al., 2005) has shown that the cortex is involved in steady-state walking. This suggests that the addition of SSC features of EEG will enrich gait information contained in sEMG features and improve the accuracy of gait phase recognition. However, the difference in gait phase recognition between case 1 and case 2 and between case 2 and case 3 was significant (*p* = 0.034, *p* = 0.003) only at the lowest speed (1.4 km/h), although the accuracy of gait phase recognition increased using case 2 at speeds of 2.0 and 2.6 km/h. This may be because relatively faster walking speeds reduced sensorimotor beta-band power (Nordin et al., 2019a), and this indicates that sensorimotor cortices process more sensory feedback than slow walking (Pfurtscheller and Lopes Da Silva, 1999; Nordin et al., 2019b). Previous research showed that bilateral coordination decreased in slow than in fast walking (Plotnik et al., 2013). This suggests that people need to pay more attention to slow walking than fast walking (Plotnik et al., 2013). Furthermore, we speculated that the contribution of the EEG features to gait phase recognition was reduced at faster rather than slower speeds. Overall, the mean accuracy of gait recognition using case 2 was significantly higher (95.58%) than those using other cases (case 1: 93.47%, case 3: 71.90%) at 1.4 km/h. Therefore, the gait phase recognition based on case 2 is suitable for a relatively low speed of walking.

We also investigated the difference in the results of gait phase recognition among the three walking speeds based on case 1 and case 2, respectively. The Mann–Whitney *U*-test showed that the difference of the results of gait phase recognition among the three walking speeds based on case 1 and case 3 was significant (*p* = 0.015 and *p* = 0.045). However, the difference in the results of gait phase recognition among the three walking speeds based on case 2 was not significant (*p* = 0.224). Case 2 was more robust than case 1 although the difference in gait phase recognition between the two feature sets was not significant at a faster speed (2.0 and 2.6 km/h).

Gait phase recognition for seven participants using case 2 can be seen in Supplementary Figures 4–6. Gait phase recognition based on case 1 was better than that based on case 2 in certain gait phases. For instance, gait phase recognition based on case 1 was better than that of case 2 in LR for subject 1 at 2.0 km/h. This suggests a small difference in the individual’s level of cortex participation during walking. Case 3 performed poorly, although case 2 achieved accurate gait recognition (Figure 4). This demonstrates that gait phase information weakened when combining sEMG features with MPF of EEG. MPF is a kind of frequency domain feature. Lee et al. (2019) reported that time domain features achieved the highest accuracy than the frequency domain and common spatial patterns in multiclass MI. Therefore, we speculated that the MPF of EEG is not suitable for fusing with sEMG features to identify the gait phase.

Cortical activation is time-locked to the gait cycle, as has been demonstrated by several researchers (Gwin et al., 2011; Kline et al., 2016; Artoni et al., 2017). However, leg muscles drive leg movement directly, while corticomuscular interaction analysis in a high level of gait phase granularity was not investigated in the time–frequency domain. The latter is critical for the rehabilitation of gait disorders, especially non-traumatic gait disorders. By computing TFCMI values between EEG and sEMG using beta-band EEG data and sEMG data (Figures 5–8), we found that the distribution of TFCMI values of eight topographies (eight muscles) was different at PSW (*p* < 0.05) and TSW phases (*p* < 0.05), which indicates that the cerebral cortex area is more actively involved in the regulation of eight muscles during PSW and TSW. Additionally, similar cortex areas were activated in the eight muscles at other gait phases. Previous authors have suggested that corticomuscular connectivity was stronger in the muscles of swing legs than those of stance legs (Artoni et al., 2017). These findings do not contradict ours. The PSW and TSW gait phases are the beginning and end of swing phases, respectively, therefore, our study also showed the corticomuscular connectivity was more active in the swing phases than in the stance phases. The different cerebral cortex areas are involved in the regulation of eight muscles during PSW and TSW. However, the recognition accuracies of PSW (97.05%) and TSW (94.97%) based on case 2 were lower than other phases at the speed of 2.0 km/h. One explanation is that the different distribution of TFCMI values of eight topographies (eight muscles) reduces the separability of sEMG and EEG features. For example, in Supplementary Figure 7, there is no clear line of the SSC of sEMG and EEG at PSW and TSW.

For each muscle, corticomuscular interaction analysis for the seven gait phases has not been previously investigated in the time–frequency domain using the TFCMI method. However, are the differences in TFCMI values significant in the seven gait phases? We calculated TFCMI values for the frontal, central, and parietal lobes, and the results can be seen in Figure 9 (beta-band EEG signals). We found that TFCMI value differences between LR and MST and between TST and PSW of each muscle were not significant (Table 1). Therefore, the differences in corticomuscular interaction between LR and MST and between TST and PSW were not significant. This can be proven by gait recognition results based on case 3. The *post-hoc* tests of the gait phase recognition results based on case 3 showed that the differences of recognition results between LR and MST (*p* = 0.594) and between TST and PSW (*p* = 0.191) were not significant. The TFCMI results also can be used to explain the bad performance of the recognition results of TST and PSW based on case 3 at the speed of 2.0 km/h.

Based on this study, a high level of gait phase granularity recognition during treadmill walking by EEG and sEMG signals of the participant is available. This can be applied to the control system of a gait rehabilitation device for people with gait disorders. The gait phase recognition based on case 2 is suitable for patients with a relatively low speed, and based on case 1, case 2, and case 3, it is suitable for patients with a relatively high speed. Also, case 2 was more robust than case 1 and case 3. The cerebral cortex area is more actively involved in the regulation of eight muscles during PSW and TSW. Therefore, targeted rehabilitation training for PSW and TSW will improve the function of the gait-related cortex. Furthermore, there is no clear line of the SSC of sEMG and EEG at PSW and TSW; thus, case 2 is not suitable for gait phase recognition at the speed of 2.0 km/h. The differences of corticomuscular interaction between LR and MST and between TST and PSW were not significant. The performance of the recognition results of TST and PSW based on case 3 at the speed of 2.0 km/h was poor. Therefore, other strategies should be used to classify TST and PSW at the speed of 2.0 km/h. Our results can guide rehabilitation physicians as they develop a rehabilitation plan for each phase of a patient’s gait.

## Data Availability Statement

The raw data supporting the conclusions of this article will be made available by the authors, without undue reservation.

## Ethics Statement

The studies involving human participants were reviewed and approved by the Institutional Review Boards of Xi’an Jiaotong University. The patients/participants provided their written informed consent to participate in this study.

## Author Contributions

PW designed the study, conducted, supervised the experimental process, analyzed the experimental data, and wrote this manuscript. JZ guided the experimental process. BW co-designed the study. JH supervised the data analysis. All authors revised and approved the manuscript.

## Conflict of Interest

The authors declare that the research was conducted in the absence of any commercial or financial relationships that could be construed as a potential conflict of interest.

## Footnotes

1 www.seniam.org
