# Supplementary figures and images for: Surface Electromyography and Electroencephalogram-Based Gait Phase Recognition and Correlations Between Cortical and Locomotor Muscle in the Seven Gait Phases
Source: Front Neurosci. 2021 May 21;15:607905. doi: 10.3389/fnins.2021.607905 (PMC8175803; doi:10.3389/fnins.2021.607905)

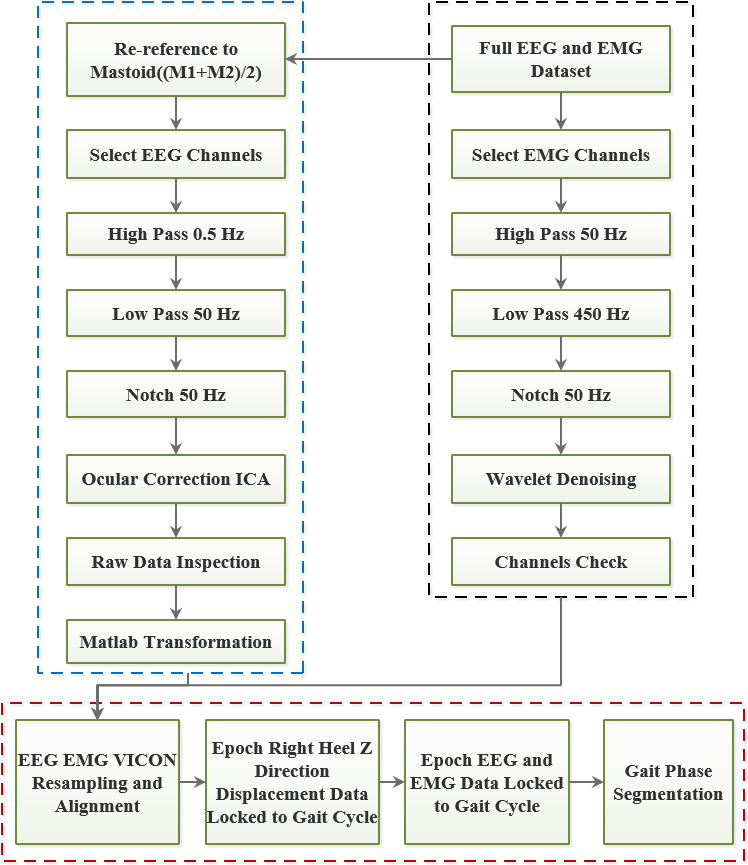

Supplement: Supplementary file 1 [file Image_1.TIF]

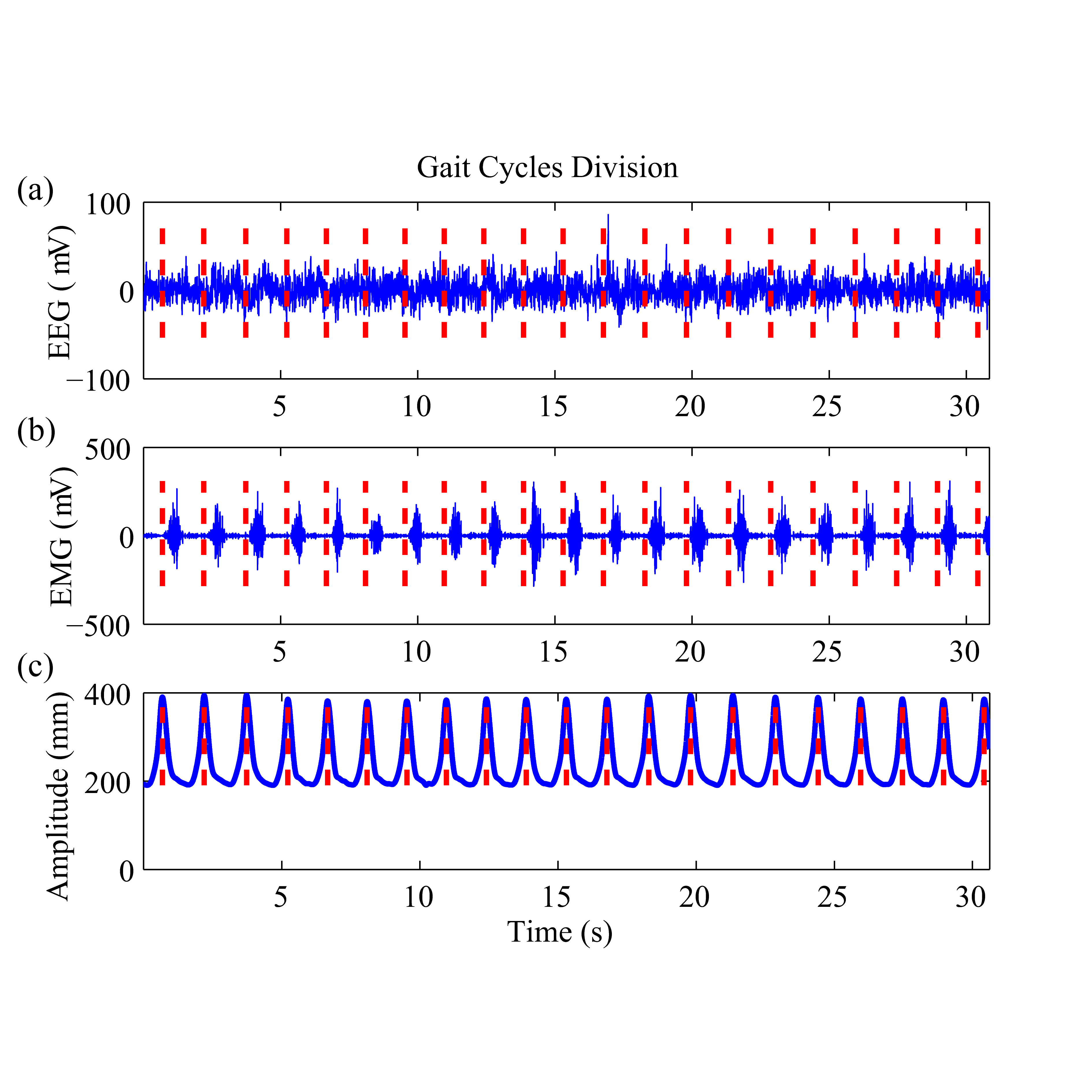

Supplement: Supplementary file 2 [file Image_2.TIF]

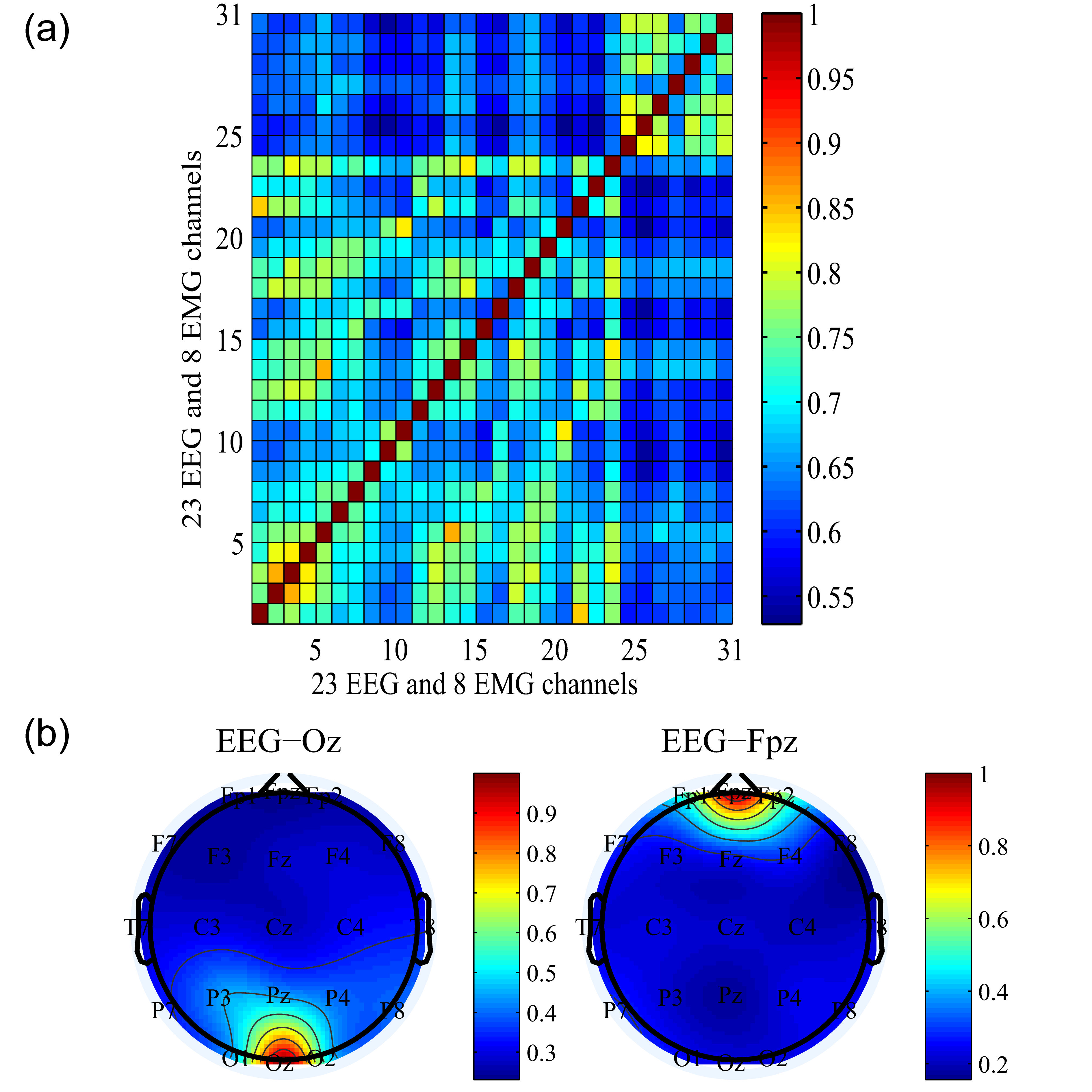

Supplement: Supplementary file 3 [file Image_3.TIF]

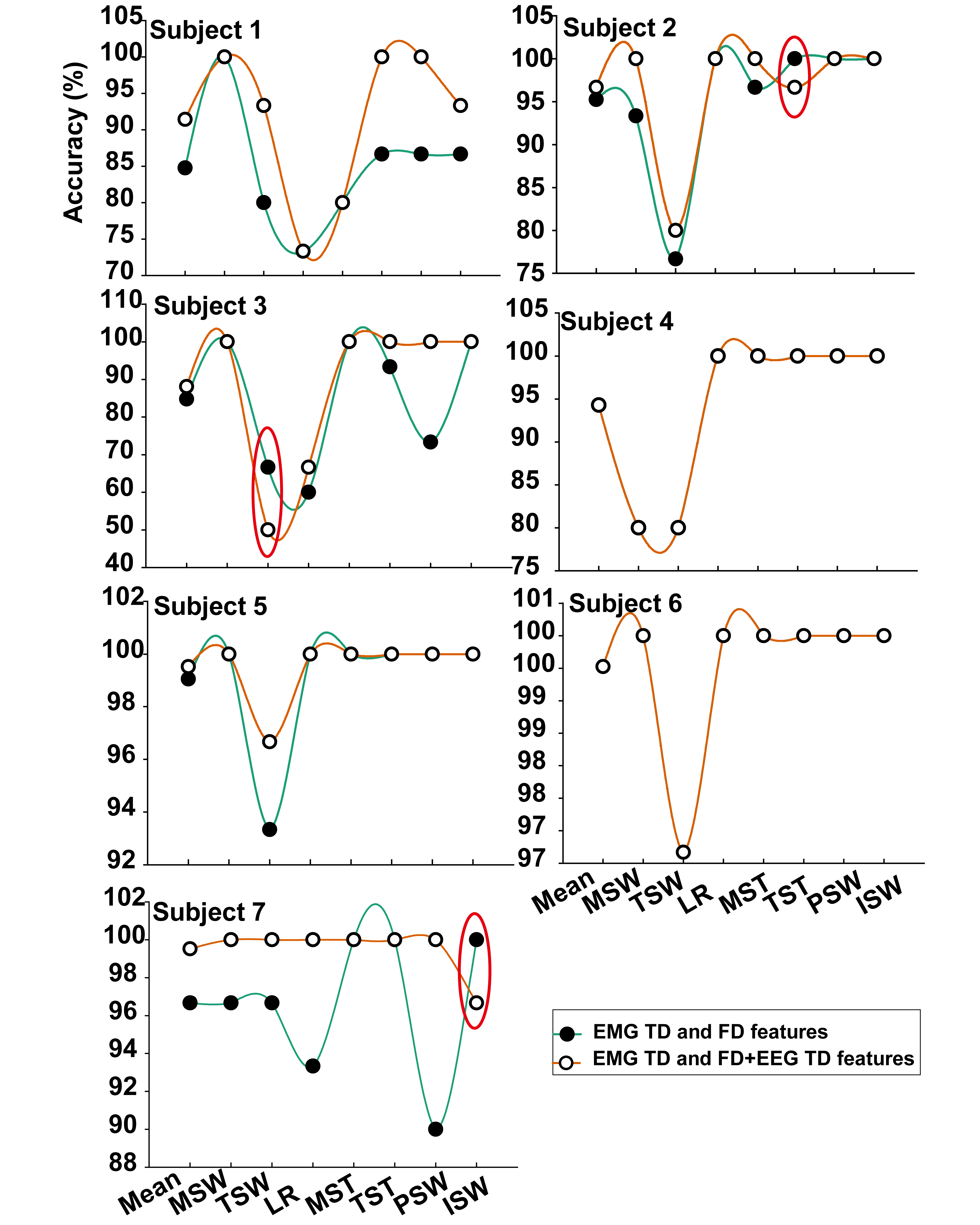

Supplement: Supplementary file 4 [file Image_4.TIF]

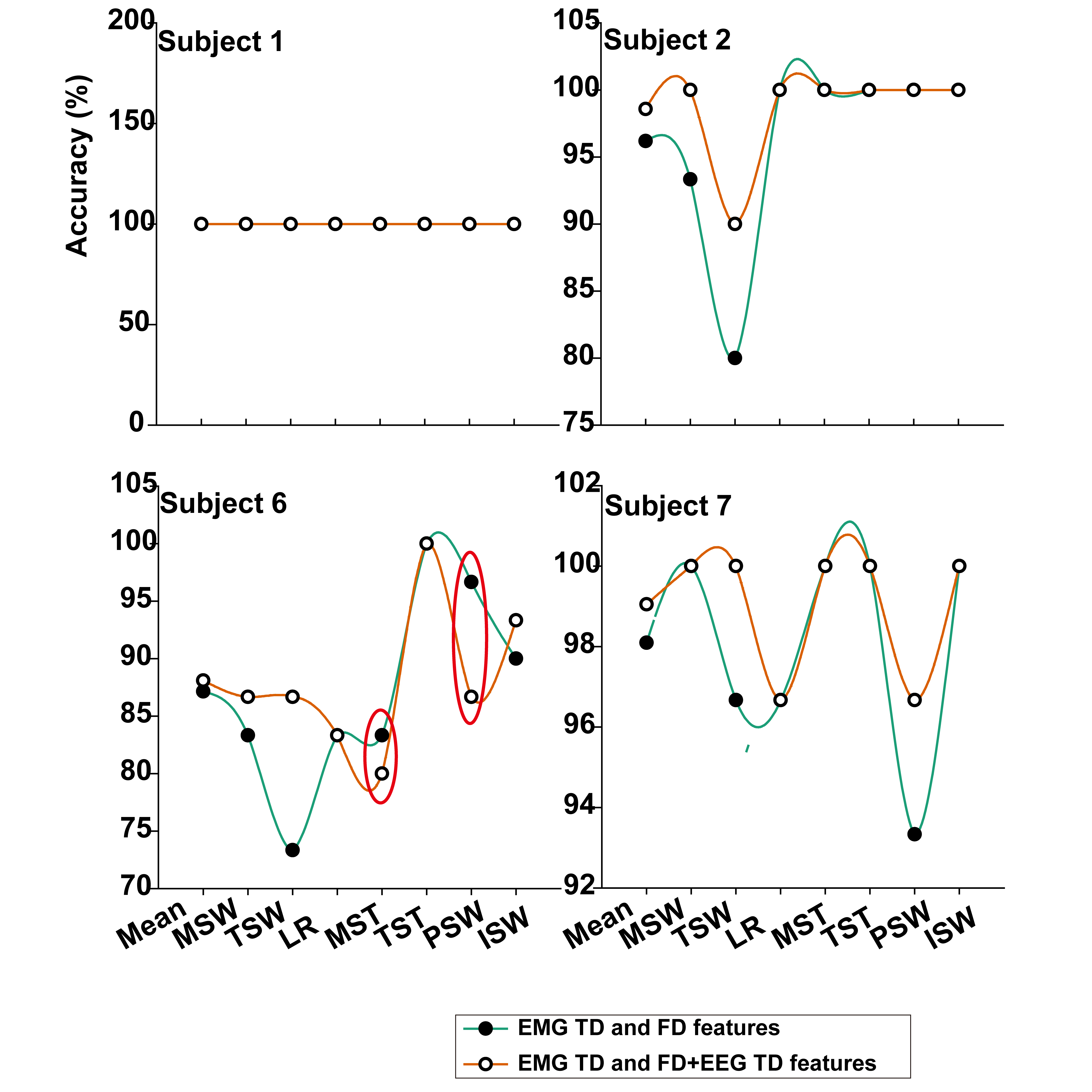

Supplement: Supplementary file 6 [file Image_6.TIF]
